# Altered functional connectivity associated with time discounting in chronic pain

**DOI:** 10.1038/s41598-019-44497-5

**Published:** 2019-05-31

**Authors:** Kenta Wakaizumi, Rami Jabakhanji, Naho Ihara, Shizuko Kosugi, Yuri Terasawa, Hiroshi Morisaki, Masao Ogaki, Marwan N. Baliki

**Affiliations:** 1 Department of Anesthesiology, Keio University School of Medicine, Tokyo, Japan; 2 Faculty of Economics, Keio University, Tokyo, Japan; 3 Shirley Ryan AbilityLab, Chicago, Illinois USA; 4 Department of Physical Medicine & Rehabilitation, Northwestern University Feinberg School of Medicine, Chicago, Illinois USA; 5 Department of Physiology, Northwestern University Feinberg School of Medicine, Chicago, Illinois USA; 6 Department of Psychology, Keio University, Tokyo, Japan

**Keywords:** Chronic pain, Reward

## Abstract

Chronic pain (CP) is a global problem extensively associated with an unhealthy lifestyle. Time discounting (TD), a tendency to assign less value to future gains than to present gains, is an indicator of the unhealthy behaviors. While, recent neuroimaging studies implied overlapping neuro mechanisms underlying CP and TD, little is known about the specific relationship between CP and TD in behavior or neuroscience. As such, we investigated the association of TD with behavioral measures in CP and resting-state brain functional network in both CP patients and healthy subjects. Behaviorally, TD showed a significant correlation with *meaningfulness* in healthy subjects, whereas TD in patients only correlated with pain intensity. We identified a specific network including medial and dorsolateral prefrontal cortex (PFC) in default mode network (DMN) associated with TD in healthy subjects that showed significant indirect mediation effect of *meaningfulness* on TD. In contrast, TD in patients was correlated with functional connectivity between dorsolateral PFC (DLPFC) and temporal lobe that mediated the effect of pain intensity on TD in patients. These results imply that TD is modulated by pain intensity in CP patients, and the brain function associated to TD is shifted from a medial to lateral representation within the frontal regions.

## Introduction

Chronic pain (CP), a persistent unpleasant sensory and emotional experience, is one of the biggest global burdens. Prevalence rate of CP is at least 20 to 40% in each country^1–10^, and health care and lost productivity cost is estimated to be up to hundreds of billion dollars per year^11–14^. In the United States, the socio-economic cost of CP was larger than the ones of other costly major diagnoses including cardiovascular diseases, neoplasms, nutritional and metabolic diseases, and respiratory system diseases^11^.

CP is strongly associated with an unhealthy lifestyle^15^. CP and obesity adversely impact each other, and weight loss for obese pain patients is an important aspect of pain rehabilitation^16,17^. Smoking in subacute low back pain patients is a risk factor of persistent pain^18^, and smokers have a lower pain threshold and experience more pain than non-smokers and former smokers^19,20^. Inactivity and fear of movement is independently associated with low back pain, joint problems, and neck and shoulder pain^21–24^. In particular, sedentary behavior is an important therapeutic target and exercise is a main approach in CP management. Regular exercise programs including the cognitive behavioral approach can attenuate chronic pain and improve patients’ lifestyle^25–27^. Addressing the change of lifestyle including exercise behavior in CP patients is a first-line intervention to reduce the socioeconomic burden related to chronic pain as well as other non-communicable diseases^28,29^.

Time discounting (TD), a tendency to assign less value to future gains than to present gains, is an indicator of unhealthy behavior. Higher TD is a significant risk factor for unhealthy diets, overweight and obesity, and lower TD was associated with greater weight reduction after a treatment^30^. Smokers also discounted the future more than non-smokers and, in longitudinal studies, higher TD predicted future smoking, as well as smokers with lower TD rates achieved higher quit rates^31^. Additionally, higher TD is a common feature of substance use including opioids and other addictive disorders^32,33^. Opioid abuse and addiction is another problem on the treatment of chronic pain^34^. Exercise behavior, a goal of CP management, is associated with lower TD rate in older adults^35^. While these findings show that CP and TD exhibit overlapping associations with various behavioral aspects of unhealthy lifestyles, the direct relationship between TD and chronic pain characteristics remain unexplored.

In accordance with the behavioral associations discussed above, brain imaging studies showed TD and CP map to similar brain regions. Subjective value of delayed monetary rewards is assessed in medial prefrontal cortex (MPFC), ventral striatum, and posterior cingulate cortex (PCC). Activity in these brain regions increases as the objective amount of a reward increases, and decreases as the imposed delay to a reward increases^36^. Nucleus Accumbens (NAc), or ventral striatum, is a key component of the brain reward system, and mesocorticolimbic system including NAc and MPFC works for reward processing and decision making^37^. On the other hand, CP makes a significant change of functional network in NAc and MPFC of patients^38^. Furthermore, resting state functional connectivity (RSFC) between NAc and MPFC in back pain patients is involved in the development of persistent pain from the subacute condition^39^. Despite the similarities of unhealthy lifestyle and brain function associated with TD and CP, little is known about the relationship of them in behavior or neuroscience. This study investigated the association of TD with behavioral measures relating to CP and resting-state brain network both in CP patients and healthy subjects.

## Results

### Association of time discounting factor to pain intensity in chronic pain patients

**Figure 1:**
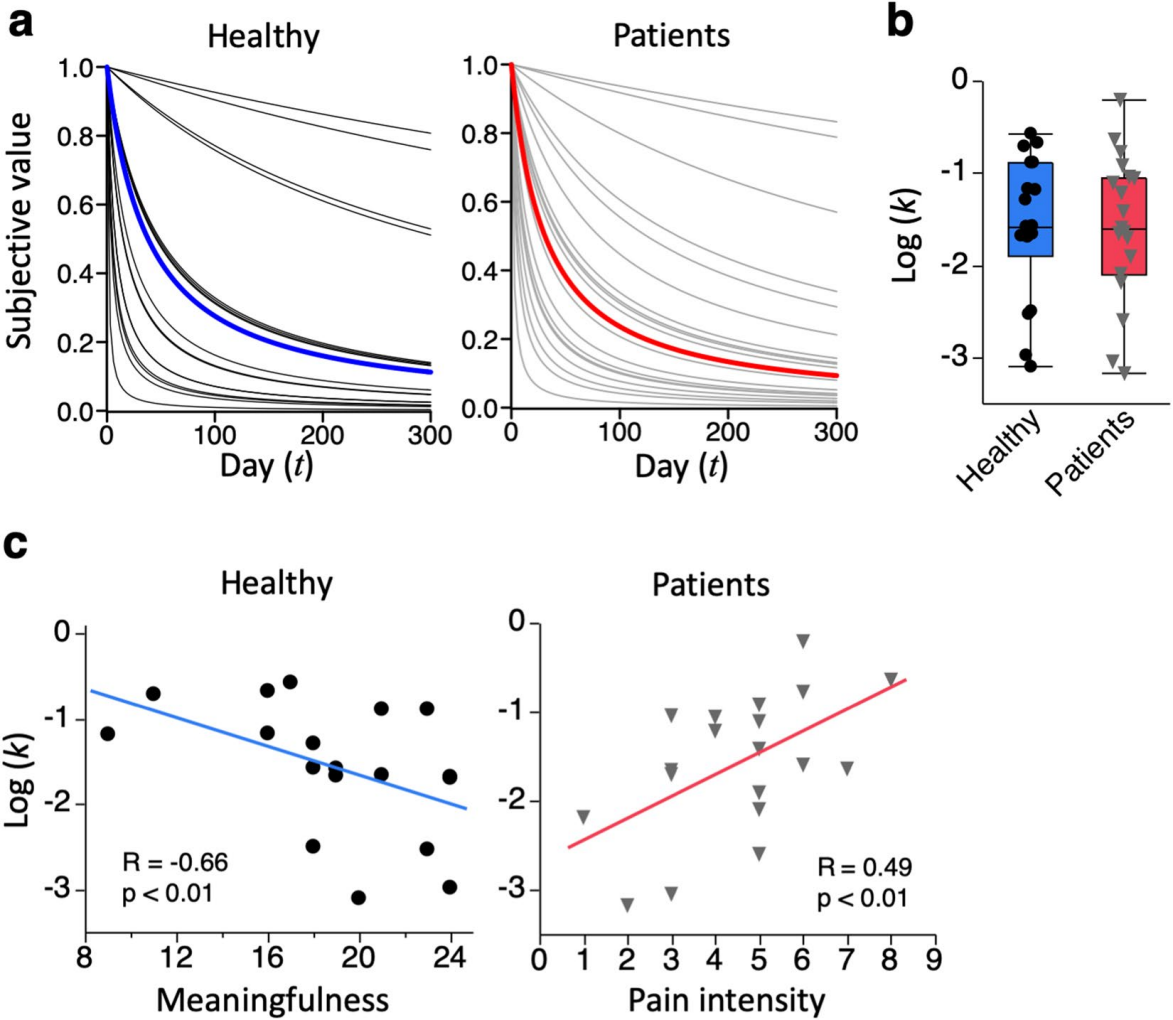
Time discounting in healthy and patients. (**a**) Plots display individual time discounting functions for healthy subjects (left) and patients (right). Blue and red line represents mean for healthy and patients respectively. **(b)** Box plot shows log-transformed discounting factor ‘*k*’ for healthy (blue) and patients (red). There was no significant difference between healthy and patients (p = 0.74). **(c)** Scatter plots show the correlation between behavioral measures and time discounting. In healthy subjects (left), time discounting show significant correlation with *meaningfulness*. In contrast, time discounting only showed correlation with pain intensity in patients (right). R = correlation coefficient.

We compare and contrast TD in a group of CP patients and matched healthy subjects and its relationship to various behavioral and clinical parameters including pain, anxiety, depression, financial strain and several Sense of Coherence (SOC) questionnaire such as comprehensibility, manageability, meaningfulness. TD was assessed by fitting a hyperbolic discounting model (1), a well validated assumption of TD in time inconsistency^36,40^, to the data (Fig. 1a). We estimated a discount function of subjective values, SV(*t*), for each subject.

1 $$SV(t)=\frac{1}{1+kt}$$

where *t* is the delay in days from the reference time point, and *k* (>0) is the time discount factor. For large values of *‘k’*, subjective value drops rapidly with time; whereas when *‘k’* is small, subjective value exhibits a slower response. In other words, subjects with small *‘k’* are more patient and less impulsive than subjects with larger *‘k’* values (Supplementary Fig. 1). Behavioral questionnaires of anxiety and depression, insomnia, and fear for movement recorded significantly higher scores in CP patients compared to healthy subjects as previously reported^24,41,42^ (Table 1). Estimated discount factor ‘*k*’ (Fig. 1b), 3 subscales of SOC questionnaire (*comprehensibility*, *manageability*, *meaningfulness*), and financial strain had no significant differences between the 2 groups. However, generalized regression analyses revealed that only *meaningfulness* significantly correlated with TD in healthy subjects, whereas, in patients, only pain intensity significantly correlated with TD (Fig. 1c, Supplementary Tables 1 and 2).

**Table 1:** Time discounting and clinical behavioral differences between in healthy subjects and CP patients.

	Healthy (n = 19)	Patients (n = 19)	p-value
	(mean, SEM)	(mean, SEM)	
log (k)	−1.49, 0.19	−1.58, 0.18	0.74
Pain intensity (1–10)	—	4.53, 0.40	
K6	1.16, 0.41	6.05, 1.25	**<0.01**
AIS	2.79, 0.58	6.47, 0.84	**<0.01**
TSK	15.79, 1.33	27.00, 1.01	**<0.01**
Comprehensibility	22.84, 1.67	20.42, 1.55	0.29
Managebility	18.47, 0.76	16.00, 1.16	0.12
Meaningfulness	18.89, 0.95	17.53, 1.36	0.42
	(n, %)	(n, %)	
High financial strain	7, 36.8	6, 31.6	0.74

Time discounting factor ‘*k*’ showed no significant difference between patients and healthy. K6, AIS, and TSK were significantly high in patients compared to healthy. SEM = standard error of mean; K6 = Kessler Psychological Distress Scale; AIS = Athens Insomnia Scale; TSK = Tampa Scale for Kinesiophobia. Age and gender adjusted regression analyses were performed for continuous data, and age and gender adjusted logistic analysis was performed for categorical data.

### Brain functional differences between CP patients and healthy

**Figure 2:**
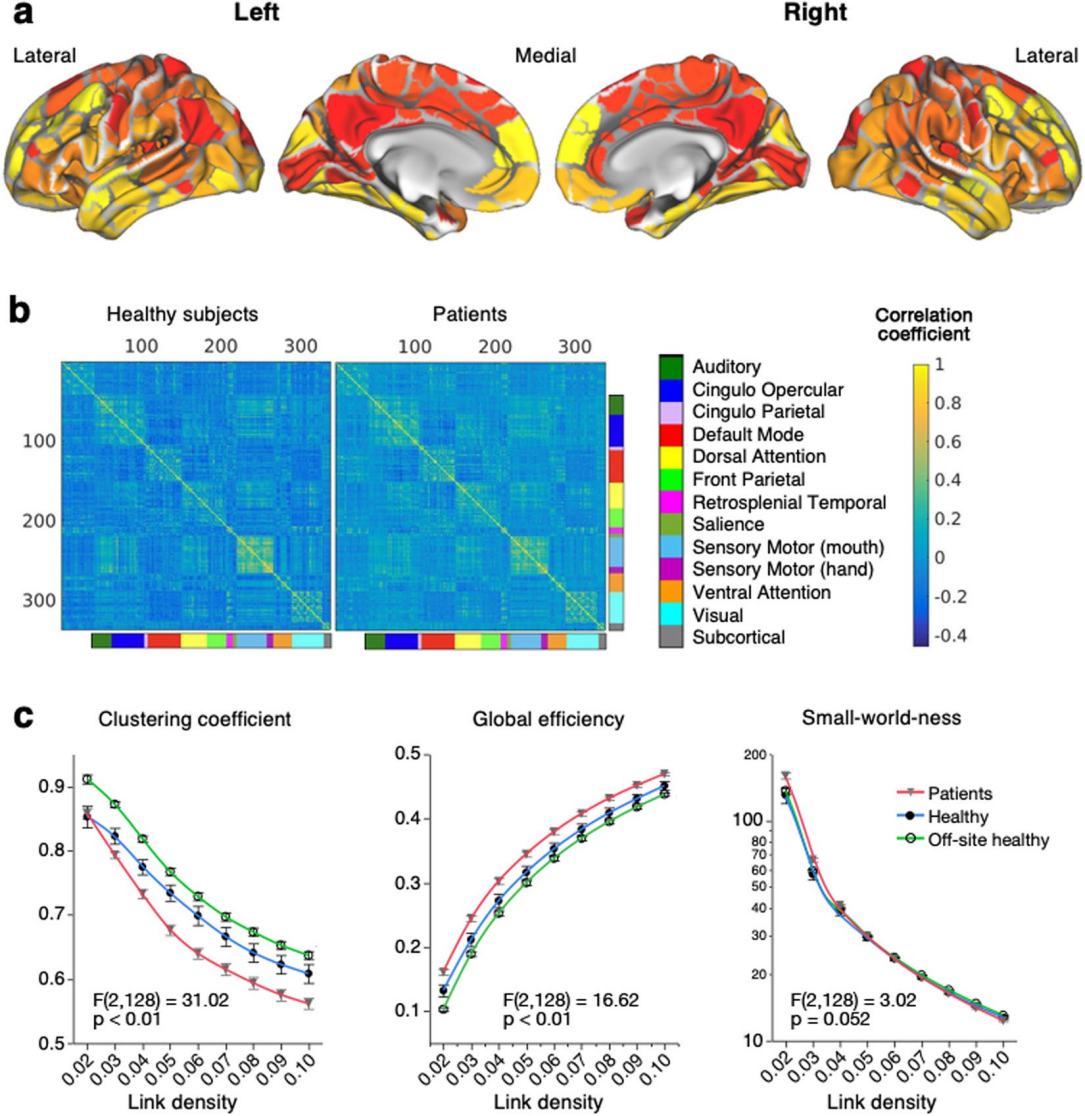
Construction and characterization of functional brain networks in healthy subjects and patients. (**a**) Whole brain atlas for regions of interest (ROIs). Ten subcortical regions from Harvard-Oxford Atlas^44–47^ were added on the 333 validated parcels derived from boundaries of resting-state functional connectivity (RSFC)^43^. **(b)** Functional connectivity matrices of the validated ROIs and 13 communities. The color bar shows the intensity of correlation coefficient. **(c)** Global graph properties in healthy (n = 19), patients (n = 19), and off-site healthy subjects (n = 95). Clustering coefficient and global efficiency were significantly different between in healthy and patients, whereas small-world-ness was not different; repeated measure ANCOVA.

We assessed functional connectivity on the predetermined 333 cortical parcels derived from boundaries of resting-state functional connectivity (RSFC)^43^ and 10 subcortical regions extracted from the Harvard-Oxford Atlas^44–47^ (Fig. 2a). The 343 regions on interest (ROIs) had been assigned to 13 predetermined community networks; auditory (Au), cingulo opercular (CiO), cingulo parietal (CiP), default mode (DM), dorsal attention (DA), front parietal (FP), retrosplenial temporal (RT), salience (Sa), sensory motor (SM) of mouth, sensory motor (SM) of hand, ventral attention (VA), visual (Vi), and subcortical (SC). For the robustness of our brain functional data, standardized graph theoretical method was demonstrated in CP patients, in our healthy subjects, and in 95 age- and gender-matched off-site healthy subjects, taken from NITRC database as reported previously^38^ (Fig. 2b). Clustering coefficient, global efficiency, and small-world-ness showed similar trends in the 3 groups, but clustering coefficient significantly decreased and global efficiency increased in CP patients compared to healthy subjects according to each link density, which is a threshold based on percentage of links with higher correlation (Fig. 2c).

### Involvement of the default mode network to time discounting in healthy

**Figure 3:**
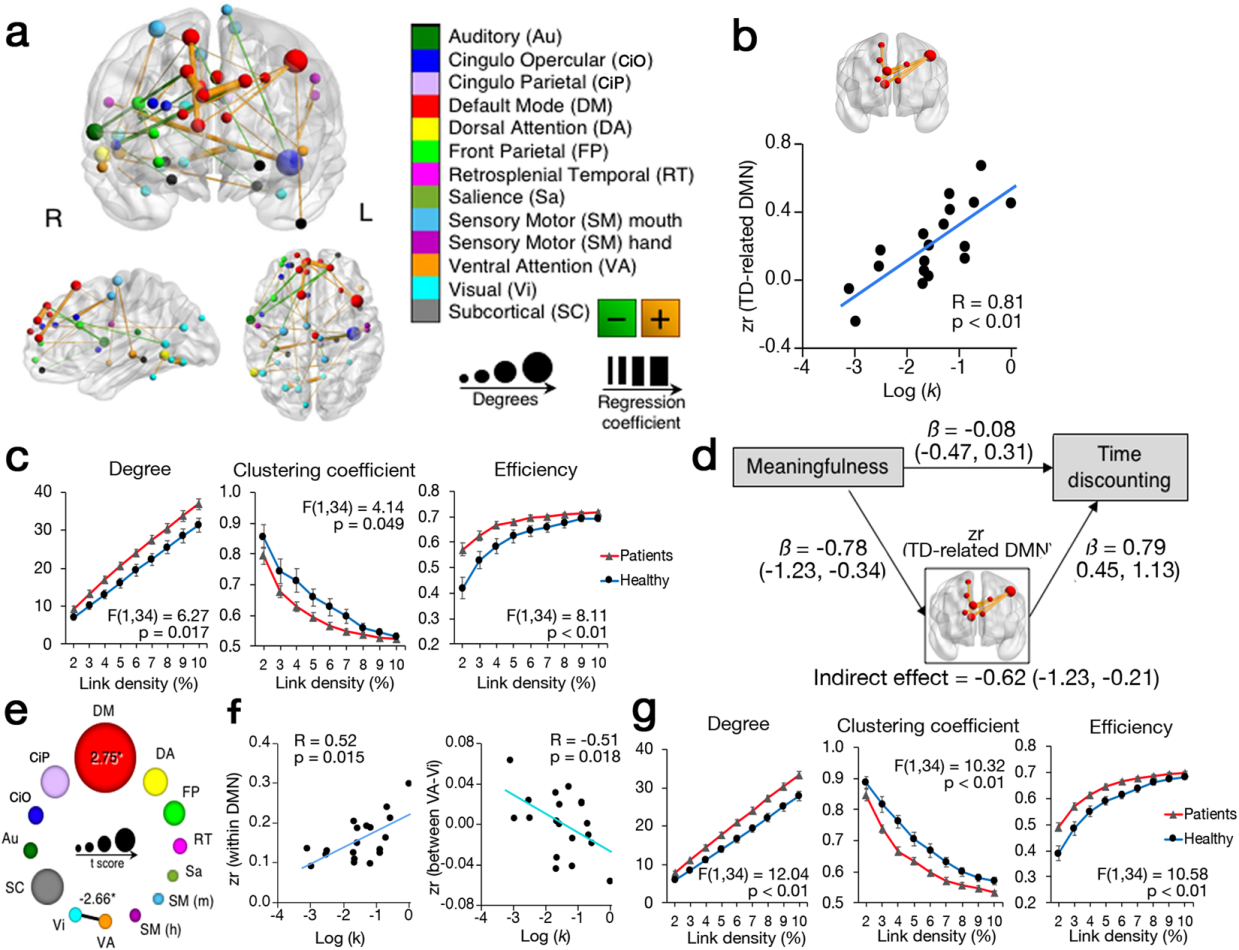
Functional connectivity associated with time discounting in healthy subjects. (**a**) Significant positive networks associated with time discounting (TD) factor in whole brain analysis (pFDR < 0.05 and cluster criterion ≥2 links). Color and size of spheres represent community membership and the number of links (degrees), respectively. Color and width of links represent correlation value and intensity (negative correlations are green; positive correlations are orange). **(b)** Brain image show DMN specific connectivity significantly associated with TD. Scatter plot shows the relationship between TD and average functional connectivity within DMN connections. **(c)** Graph metrics of the DMN specific connectivity network. Patients showed significantly higher degree and efficiency, and lower correlation coefficient through 2% to 10% link densities. **(d)** Mediation analysis of the pathway from the meaningfulness to the log-transformed TD factor. Standardized regression coefficient ‘β’ was shown with 95% confidence interval (**p < 0.01). Indirect effect was computed in bootstrap method permuted 10000 times. **(e)** Schematic result of the community-based regression analysis for TD. Only the connection within DMN and between VA and Vi networks showed the significant correlation to the log-transformed TD factor. **(f)** Scatter plots show association of TD with the whole connection within DMN and the network between VA and Vi. **(g)** Graph metrics of the whole DMN. Patients showed significantly higher degree and efficiency, and lower correlation coefficient through 2% to 10% link densities. zr = Fisher’s z-transformed regression coefficient. All statistical analyses were controlled with age and gender.

ROI-based connectivity analysis identified 45 links involving 51 ROIs related to TD, in which each RSFC cluster met a criterion of at least 2 links connected with each other and every link showed less than 0.05 false discovery rate-adjusted p-value (pFDR) (Fig. 3a). When we categorized each link to the predetermined 13 communities, the greatest number of links (6 links of 8 ROIs) were categorized to the DMN. The average of the Fisher’s z-transformed correlation coefficients (zr value) of those 6 links are significantly correlated with log (*k*) (Fig. 3b, Supplementary Table 3). Number of links (degree), clustering coefficient, and efficiency were averaged in the identified 8 ROIs. Repeated measure ANOVA resulted in significant higher degree and efficiency, and lower clustering coefficient in patients compared to healthy (Fig. 3c). However, none of them were associated with log (*k*) nor *meaningfulness* across all link densities (Supplementary Table 4). Mediation analysis resulted in a significant indirect effect of *meaningfulness* on TD through the identified 6 links within the DMN (Fig. 3d, Supplementary Table 5). Averaged zr value was computed within each community and between any two communities. Increased log (*k*) was significantly associated with increased zr value within the whole DMN including 41 ROIs and decreased zr value between the VA network (23 ROIs) and the Vi network (39 ROIs) (Fig. 3e,f). The differences of graph metrics between healthy and patients are same in the whole DMN as in the TD-related DMN (Fig. 3g).

### Involvement of the dorsolateral prefrontal cortex to time discounting in CP patients

**Figure 4:**
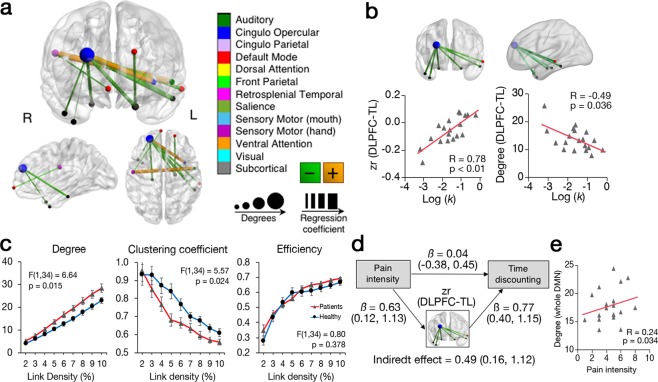
Functional connectivity associated with time discounting in chronic neck pain patients. (**a**) Significant positive networks associated with time discounting (TD) factor in whole brain analysis (pFDR < 0.05 and cluster criterion ≥2 links). Color and size of spheres represent community membership and the number of links (degrees), respectively. Color and width of links represent correlation value and intensity. **(b)** Association of the TD factor to brain functions of the identified network of right DLPFC to temporal lobe (TL). TD factor significantly associated with zr value and degree of the DLPFC-TL in the 5% link density. **(c)** Graph metrics of the DLPFC-TL network. Patients showed significantly higher degree and lower correlation. **(d)** Mediation analysis of the pathway from pain intensity to the log-transformed TD factor. Standardized regression coefficient ‘β’ was shown with 95% confidence interval. Indirect effect was computed in bootstrap method permuted 10000 times. **(e)** Association between pain intensity and degree of the whole DMN in the 5% link density. zr = Fisher’s z-transformed regression coefficient. All statistical analyses were controlled with age and gender.

On the other hand, 10 links and 12 ROIs related to TD were identified in CP patients (pFDR < 0.05 and cluster criterion ≥ 2 links), and no overlap was observed with the links in healthy subjects (Fig. 4a). We focused on the right dorsolateral prefrontal cortex (DLPFC) included in the CO network, because the greatest number of connections (7 links) were in the discovered network region. The DLPFC mainly connected to the temporal lobe (TL) negatively, which covered the bilateral Parahippocampal gyrus (Supplementary Table 6). The increased zr value and decreased degree significantly associated with increased log (*k*) (Fig. 4b, Supplementary Table 7). Moreover, patients showed significant higher degree and lower clustering coefficient compared to healthy (Fig. 4c). Mediation analysis resulted in significant indirect effect of the pain intensity on TD through the coupling of DLPFC to TL (Fig. 4d, Supplementary Table 8). Furthermore, pain intensity was associated with the degree of the whole DMN (Fig. 4e, Supplementary Table 7). Regarding involvement of anxiety and depression in the TD factor and brain functions associated with pain intensity, multiple regression analyses were performed including Kessler Psychological Distress Scale (K6), and model fitting values were compared to the model of pain intensity (pain model). Any models with K6 for log (k), zr value of DLPFC-TL, and degree of the whole DMN showed decreased F-value and adjusted R^2^, increased small-sample-size corrected version of Akaike information criterion (AICc) and Bayesian information criterion (BIC) compared to the pain model (Supplementary Table 9).

Finally, we hypothesized that DMN worked on a 3-step mediation model from pain intensity to TD and tested it. The model represents a series of mediation pathway composed of a few brain networks (Supplementary Fig. 3). The TD-related DMN identified in healthy and the whole connections within DMN were put into the box “A” and “B” in the models respectively, as well as every connection within DMN were tested one by one. However, none of them showed significant fitting on these models (Supplementary Table 10).

## Discussion

Our findings identified a significant correlation of pain intensity to TD in CP patients. Patients with severe pain showed high discount rate for future value. This finding implies that severe pain will indicate low expectancy for future condition. Eventually, patients with intractable chronic pain have unfavorable cognitions regarding the probability of future experiences, events, and behavior^48^. Therefore, pain intensity is supposed to increase TD and influence an individual’s decision making of inter-temporal choices, even if they do not directly relate to pain. In contrast, *meaningfulness*, a subscale of SOC, correlated with TD in healthy subjects, whereas it was not replicated in patients. This suggests that pain is a more powerful influencer of TD than *meaningfulness* in chronic pain state. Since CP state shows functional changes of brain neurons, neuro transmitters, receptors, and neural circuits, especially in the brain reward system^49,50^, this neural plasticity may contribute to the different mechanisms of the intertemporal decision-making between healthy and CP patients.

TD variability in patients might contribute to compliance of pain treatments. Higher TD patients will prefer standard pain treatments, such as drug administrations and injection therapy, which can provide their effects within from a few hours to a few days. On the other hand, advanced interdisciplinary pain programs like a cognitive behavioral therapy (CBT) are probably not acceptable for them, because they will need at least a few weeks as a window period to recognize pain modulation. Since negative RSFC of the DLPFC-TL was associated with lower TD in the current study, the negative coupling of the DLPFC-TL may be a potential predictor of successful pain programs. Furthermore, amending higher TD may be an initial therapeutic target for interdisciplinary pain treatment and the DLPFC-TL may be helpful as a biomarker.

DMN including MPFC and PCC is a brain network shown to be active in resting-state^51,52^. In healthy subjects, community-based analysis revealed that TD correlated with RSFC of the whole DMN. MPFC and PCC were brain regions activated by assessment of subjective value^36^. Increased RSFC within a module including MPFC, DLPFC, and PCC was associated with high *impulsivity*^53^, which is a major personality shaping TD^54,55^. ROI-based analysis identified that TD-associated links in the DMN were composed of DLPFC and MPFC (Supplementary Table 3); that is consistent with previous findings^56,57^. In addition, the increased RSFC of the specific links within DMN represented a negative correlation to *meaningfulness*. According to a previous study, increased RSFC of the DMN is associated with lower levels of happiness^58^. Furthermore, our result of mediation analysis showed the specific DMN is a mediator on the effect of meaningfulness on TD.

Similar to the behavioral results of the TD-related measures in CP patients compared to healthy, we identified completely different connections associated with TD in patients. Both DLPFC and TL are common brain areas involved in decision making and reward processing^37^. The coupling of DLPFC-TL, however, has never been identified as a TD-related region in healthy. As many previous studies revealed, chronic pain dramatically alters the functional architecture of brain^38,59–66^. Indeed, graph theoretic analyses revealed significant differences of global and local brain function between patients and healthy in this study (Figs 2c, 3c,g, 4c). Furthermore, the mediation effect of the DLPFC-TL on the relationship between pain intensity and TD implies that the functional change of the brain precipitated by CP consequently makes the DLPFC-TL acquire the function related to TD. In other words, as degree of the whole DMN was associated with pain intensity, chronic painful condition makes the impulsive system involved in TD shift from DMN to the coupling of the DLPFC-TL.

Previous literatures identified higher TD in depressive people^67,68^. Since depression is also a confounding factor of pain^69,70^, it may involve to the association of pain to the increased TD and the TD-related brain functions. However, any multiple regression models including K6 did not show improvement of the models’ fitting in terms of TD factor and TD-associated brain functions (Supplementary Table 9). This means that anxiety and depression are less effective on TD than pain intensity in CP patients.

Small number of subjects may cause a selection bias for the current study. Unfortunately, this is the first study regarding TD in CP patients so that there is no brain study to discuss the reliability of our findings. However, it can be expected to be a stable result, as our findings in healthy were consisted with previous evidences.

Since TD data in this study was gathered by a monetary questionnaire, magnitude effect, an amount-dependent discounting, was not controlled. Magnitude effect is a phenomenon that small rewards are discounted more than large ones, and the anomaly is commonly reported in literature^71^. Subjective differences in feeling about the amount of money offered might have influenced outcomes in this study. Financial strain is an objective indicator of socioeconomic status^72^ and significantly correlates with income^73^. In the present study, TD factor was indifferent between high and low financial strain people both in healthy (mean, standard error = -1.4, 0.3 vs. -1.5, 0.2; in high vs. low strain) and in CP patients (−1.3, 0.3 vs. −1.7, 0.2). That suggests the magnitude effect has little influence on this study.

Pain intensity likely to make the TD-related brain network shift from the DMN to the DLPFC-TL in CP patients. Although we hypothesized the functional alteration of the DMN precipitated by CP affected the new construction of the TD-related network, the 3-step mediation analyses did not show any significant results (Supplementary Fig. 3 and Table 7). From the transition of subacute to chronic pain, RSFC between Hippocampus and MPFC dramatically decreases^74^, and Parahippocampal gyrus in TL mediates the relationship between Hippocampus and chronic pain^60^. These brain interactions may be hypothesized to induce the functional shift of the neuro mechanisms underlying TD, and a subsequent longitudinal study will be required to identify that.

In conclusion, CP patients have different resting state brain networks underlying TD compared to healthy subjects. Specifically, in CP patients, the functional coupling of the DLPFC-TL precipitated by chronic pain plays a significant role in TD.

## Methods

### Subjects

We recruited 19 patients with chronic neck pain (6 males, and 13 females, mean age = 45 years, age range = 21–64 years) and 19 age- and gender-matched healthy volunteers. Specific inclusion criteria for chronic neck pain were (1) experiencing pain in the neck, (2) pain persisted for longer than the last 3 months, and (3) pain intensity of 4/10 or greater on the numerical rating scale (NRS). All participants were right-handed and underwent structural and resting-state functional magnetic resonance image (rs-fMRI) scanning. Subjects were excluded if they reported (1) history of head injury, (2) medication of epilepsy and/or depression, (3) to be unable to keep supine position, (4) contraindication of MRI, and (5) the score of 5/7 or greater on the Stanford Sleepiness Scale (SSS) performed soon after the scanning. All subjects passed radiological diagnosis for brain structural abnormality. In addition, 95 off-site healthy control subjects (30 males and 65 females, mean age = 45 years, age range = 21–65 years) were taken from NITRC 1000 functional connectomes project (http://fcon_1000.projects.nitrc.org/). The present study was approved by the Keio University Institutional Review Board committee (approval number: 20160002) and all participants accepted the written informed consent. All experiments were performed in accordance with Helsinki declaration and an ethical guideline for medical and health research involving human subjects issued by Japanese Ministry of Health, Labour and Welfare. This trial is registered with the University Hospital Medical Information Network (UMIN) clinical trials registry, number UMIN 000024475.

### Clinical measures

Participants completed self-report questionnaire including NRS for pain intensity, Kessler Psychological Distress Scale (K6), Athens Insomnia Scale (AIS), Tampa Scale for Kinesiophobia (TSK), Sense of Coherence (SOC), financial strain, and time discounting questionnaire. K6 is used for scaling anxiety and depression. TSK includes 11 items to measure fear for movement. SOC represents anti-stressful personality composed of 3 subscales, *comprehensibility*, *manageability*, and *meaningfulness*. Financial strain is rated on a 5-point Likert scale ranging from 1 (very tight) to 5 (enough), and subjects who marked “very tight” of “tight” were categorized into the high-strain group. All questionnaires were administered on the day of brain scanning.

### Time discounting factor acquisition

Subjects were asked a set of ten questions in the following format^75^:

To me, ‘receiving $ X today’ is equally as good as ‘receiving $ Y in __days,’

where X < Y. Subjects needed to wait longer to get the larger amount of money, $Y, and they filled for the longest acceptable delay (*t*) that makes the two options the same. The actual amounts of X and Y are one of $5, $10, $15, $20 and $25; the ten combinations in total. Discount function of subjective values, SV(*t*), was estimated to fit the following Eq. (2) for each subject by the hyperbolic discounting model using non-linear least-square (NLS) method in R 3.4.0 software.

2 $$\$X=SV(t)\times \$Y$$

We also tested another assumption using exponential model (3), a time consistent model^71^.

3 $$SV(t)={e}^{-ct}$$

where *c* (>0) is an exponential discount factor. However, it returned unacceptable model fitting (R square < 0) in two subjects. In the other 36 subjects, log-transformation of exponential factor *c* significantly correlated with hyperbolic factor *k*, and the R squares in both models significantly correlated each other as well (Supplementary Fig. 2). The hyperbolic model and the factor ‘*k*’ were therefore employed in the present study.

### Brain scanning parameters

Structural and functional MRI data were obtained with a 3.0-T Signa (GE Healthcare) and 8 channel phased array coil. The parameter setting of T1-weighted sagittal brain volume imaging (BRAVO) with extended dynamic range (EDR) was following: voxel size = 1 × 1 × 1 mm; inversion time (TI) = 650 ms; flip angle (FA) = 80 degrees; in-plane matrix resolution = 256 × 256; field of view (FOV) = 256 mm. rs-fMRI images were acquired in ascending order on T2-weighted gradient echo planar imaging (EPI) using array coil spatial sensitivity encoding (ASSET) with following parameters: repetition time (TR) = 2.5 s; echo time (ET) = 30 ms, FA = 80 degrees; number of slices = 40; slice thickness/gap = 3.2/0.8 mm; in-plane resolution = 64 × 64; FOV = 212 mm; number of volumes = 240.

### Preprocessing

The preprocessing of each subject’s time series of fMRI volumes was performed using the FMRIB Expert Analysis Tool (FEAT, www.fmrib.ox.ac.jk/fsl) and encompassed: Discarding the first four volumes; skull extraction using BET; slice time correction; motion correction; and high-pass and low-pass temporal filtering (0.0075 and 0.1 Hz). These included the six parameters obtained by rigid body correction of head motion, the global BOLD signal averaged over all voxels of the brain, signal from a ventricular region of interest, and signal from a region centered in the white matter were removed from the data through linear regression as non-neuronal fluctuations. All preprocessed fMRI data were registered into standard MNI space, and subsequently registered to the 343 ROIs as mentioned in the main manuscript.

### Functional connectivity analysis

Functional correlation maps and connectivity networks were produced using a well-validated method^76^. We first extracted the BOLD time series from a predetermined functional region of interest (ROI) and then computed the correlation coefficient between its time course and the time variability of all other ROIs. Correlation coefficients were converted to a normal distribution using Fischer’s z-transform. General linear model (GLM) was computed repeatedly for all connections with a regressor of log-transformed discount factor ‘*k*’ under controlling age and gender, and then false discovery rate (FDR) was generated.

### Graph theoretical analysis

As we described previously^38,77^, subject dependent threshold was calculated according to the number of edges to compare the extracted graphs. The nodes of each extracted graph comprised with the ROIs and the edges corresponding to the absolute values of correlation coefficient greater than the threshold. We chose 9 values of threshold, from a conservative threshold corresponding to 2% connection density to a lenient threshold corresponding to 10% link density, where link density is the percentage of edges with respect to the maximum number of possible edges [N × (N − 1)/2]. We computed the ‘clustering coefficient - a measure of network segregation – and the ‘global efficiency’ - a measure of the network integration into a community structure of interconnected modules – for each link density using the brain connectivity toolbox (BCT)^78^. They were defined at the nodal level and then the global average was estimated over all nodes. We also computed the ‘small-world-ness’ based on the tradeoff between clustering and efficiency for each subject. It was computed as the multiplication of the clustering ratio and the efficiency ratio to the random network, which was generated across 100 repetitions in the same number of edges and nodes in each link density^38^. Differences in topological properties between groups were computed using a repeated measure ANCOVA, with age and gender as covariates of no interest.

### Network visualization

ROIs and functional connections were visualized on a surface rendering of a human brain atlas with the BrainNet Viewer (Xia *et al*., 2013, http://www.nitrc.org/projects/bnv/)^79^. The sizes of the spheres representing their ROI strength scaled by the degrees, and the number of edges. ROIs were color-coded according to the category of the 13 consensus communities. The width of each functional connection was weighted by correlation coefficient of the BOLD signals. Positive and negative correlations were colored orange and green respectively.

### Mediation analysis

Mediation analysis was performed with PROCESS 3.0 extension in SPSS 24^80^. Indirect effect was computed in bootstrap method permuted 10000 times. The significant fitting for the hypothetic model was defined as the significant non-zeros of the effects in all pathways including the mediators. Age and gender were controlled in all regression analyses.

## Supplementary information

Supplementary information
