# Supplementary material for: Altered functional connectivity associated with time discounting in chronic pain
Source: Sci Rep. 2019 May 31;9:8154. doi: 10.1038/s41598-019-44497-5 (PMC6544657; doi:10.1038/s41598-019-44497-5)
Supplement: Supplementary file 1 — Supplementary information [file 41598_2019_44497_MOESM1_ESM.docx]

Supplementary Information

**Altered functional connectivity associated with time discounting in chronic pain**

Kenta Wakaizumi^1,2,3,4,^*, Rami Jabakhanji^3,4,5^, Naho Ihara^1^, Shizuko Kosugi^1^, Yuri Terasawa^6^, Hiroshi Morisaki^1^, Masao Ogaki^2^, Marwan N Baliki^3,4^

1. Department of Anesthesiology, Keio University School of Medicine, Tokyo, Japan.
2. Faculty of Economics, Keio University, Tokyo, Japan.
3. Shirley Ryan AbilityLab, Chicago, Illinois, USA
4. Department of Physical Medicine & Rehabilitation, Northwestern University Feinberg School of Medicine, Chicago, Illinois, USA.
5. Department of Physiology, Northwestern University Feinberg School of Medicine, Chicago, Illinois, USA.
6. Department of Psychology, Keio University, Tokyo, Japan.

* Corresponding author: [kenta.wakaizumi@northwestern.edu](mailto:kenta.wakaizumi@northwestern.edu)

**Supplementary information contains:**

- Supplementary Figures 1-3
- Supplementary Tables 1-10

**Supplementary Figure 1. Correlation of the two discount models.** We excluded 2 subjects, who showed negative number of R square in the fitting of exponential discount model. **(a)** Scatter plot of the hyperbolic discount factor ‘*k*’ and the exponential discount factor ‘*c*’ after log-transformation. The two factors significantly correlated each other. **(b)** Scatter plot of the R squares for the hyperbolic discount model versus the exponential discount model. The R squares significantly correlated between the two models.

**Supplementary Figure 2. Subject-specific discount function. (a)** Our most impulsive subject (k = 0.620). **(b)** A subject with the nearest discount rate to the median (k = 0.027). **(c)** Our most patient subject (k = 0.001).

**Supplementary Figure 3.** **Three-step mediation models of the path form the pain intensity to the log-transformed discount factor.** Each *β* represents regression coefficient of each regression model. Previously identified networks and all connections within DMN were put into the box ‘A’ and ‘B’, respectively. The significant fitting to the model was defined as all significant non-zeros of *β_1_*, *β_2_*, *β_3_*, and indirect effect of the path including both of the DLPFC-TL network and the hypothesized valuables. Indirect effect was computed in bootstrap method permuted 10000 times. All statistical analyses were controlled with age and gender.

**Supplementary Table 1**. **Relationships of behavioral parameters to discount factor in healthy subjects.** Only meaningfulness showed significant correlation to log (k) in healthy subjects. β = regression coefficient; CI = confidence interval; SEM = standard error of mean; R = correlation coefficient; **p<0.01, age and gender adjusted regression analysis.

**Supplementary Table 2.** **Relationships of behavioral parameters to discount factor in patients.** Only pain intensity showed significant correlation to log (k) in patients. β = regression coefficient; CI = confidence interval; SEM = standard error of mean; R = correlation coefficient; *p<0.05, age and gender adjusted regression analysis.

**Supplementary Table 3.** **Each link in the specific network within DMN correlated with discount factor in healthy subjects.** MNI coordinates (x, y, z) are shown in mm. Each link is represented as the combination of left and right ROIs. The t-statistics of the log (k) and the meaningfulness were calculated using age and gender adjusted regression analyses with regression coefficient of each link. Links between DLPFC and MPFC were highlighted with bold. DMN = default mode network; ROI = region of interest; DLPFC = dorsal lateral prefrontal cortex; MPFC = medial prefrontal cortex; SFG = sprafrontal gyrus.

**Supplementary table 4. Relationships between graph metrics and behavioral measures in healthy subjects.** Averages of ROIs included the TD-related DMN or the whole DMN are calculated for each graph metric through from 2% to 10% link densities. Age- and sex-adjusted repeated measure ANOVA was performed. TD = time discounting; DMN = default mode network; DLPFC = dorsolateral prefrontal cortex; TL = temporal lobe.

**Supplementary Table 5. Mediation analyses for the specific DMN relating to TD in healthy subjects.** The TD-related DMN mediated the effect of the meaningfulness on the discount factor, but it did not satisfy the reverse pathway, from the factor to the meaningfulness. Indirect effect was computed in bootstrap method permuted 10000 times. All statistic analyses were controlled with age and gender. DMN = default mode network; TD = time discounting; zr = Fisher's z-transformed regression coefficient; CI = confidence interval; SEM = standard error of mean.

**Supplementary Table 6. Each link of DLPFC to temporal lobe correlated with discount factor in patients.** MNI coordinates (x, y, z) are shown in mm. Each link is represented as the combination of left and right ROIs. The t-statistics of the log (k) and the pain intensity were calculated using age and gender adjusted regression analyses with regression coefficient of each link. Links between DLPFC and hippocampal formation were highlighted with bold. DLPFC = dorsal lateral prefrontal cortex; ROI = region of interest; TP = temporal pole; aPaHC = anterior parahippocampus; pMTG = posterior middle temporal gyrus; pITG = posterior inferior temporal gylus.

**Supplementary table 7. Relationships between graph metrics and behavioral measures in patients.** Averages of ROIs included the TD-related DMN or the whole DMN are calculated for each graph metric through from 2% to 10% link desities. Age- and sex-adjusted repeated measure ANOVA was performed. TD = time discounting; DMN = default mode network; DLPFC = dorsolateral prefrontal cortex; TL = temporal lobe.

**Supplementary Table 8. Mediation analyses for the specific DLPFC-TL network in patients.** The DLPFC-TL network mediated the effect of the pain intensity on the discount factor, but it did not satisfy the reverse pathway, from the factor to the pain intensity. Indirect effect was computed in bootstrap method permuted 10000 times. All statistical analyses were controlled with age and gender. DLPFC = dorsal lateral prefrontal cortex; TL = temporal lobe; zr = Fisher's z-transformed regression coefficient; CI = confidence interval; SEM = standard error of mean.

**Supplementary Table 9. Comparison of age- and gender-adjusted multiple regression models including K6 to the model of pain intensity (pain model) within patients.** Change (Δ) of each value from the pain model was calculated in the model of K6 (K6 model) and of both K6 and pain (Mixed model). Adj R2 = adjusted R-square, AICc = small-sample-size corrected version of Akaike information criterion. BIC = Bayesian information criterion.

**Supplementary Table 10. Involvement of DMN in the 3-step mediation analyses.** Any networks did not satisfy the 3-step mediation model A and B in the Supplementary Figure 5. Indirect effect was computed in bootstrap method permuted 10000 times. All statistical analyses were controlled with age and gender. DMN = default mode network; DLPFC = dorsal lateral prefrontal cortex; TL = temporal lobe; zr = Fisher's z-transformed regression coefficient; CI = confidence interval; SEM = standard error of mean.
